# A Eurasia-wide polyploid species complex involving 6*x Trifolium ambiguum*, 2*x T. occidentale* and 4*x T. repens* produces interspecific hybrids with significance for clover breeding

**DOI:** 10.1186/s12870-019-2030-5

**Published:** 2019-10-22

**Authors:** Warren M. Williams, Isabelle M. Verry, Helal A. Ansari, S. Wajid Hussain, Ihsan Ullah, Nicholas W. Ellison

**Affiliations:** 1 AgResearch Grasslands Research Centre, Tennent Drive, Palmerston North, 4442 New Zealand; 2 College of Sciences, Massey University, Tennent Drive, Palmerston North, 4442 New Zealand

**Keywords:** *Trifolium ambiguum*, *Trifolium occidentale*, *Trifolium repens*, White clover, Interspecific hybridization, Unreduced gametes, Clover breeding

## Abstract

**Background:**

*Trifolium ambiguum* occurs as a 2*x*, 4*x*, 6*x* polyploid series in W Asia. The 6*x* form is the most agronomically desirable, having strong rhizomatous spread and drought tolerance. These traits would be potentially very valuable if they could be transferred to white clover (*T. repens*) which is the most important agronomic clover species. However, to-date, no fertile interspecific hybrids with 6*x T. ambiguum* are available. Previously, 2*x T. occidentale* from W Europe has produced synthetic fertile hybrids with both 2*x* and 4*x T. ambiguum* and these were inter-fertile with white clover. Here we ask whether 2*x T. occidentale* can form fertile hybrids with 6*x T. ambiguum* and act as a genetic bridge to white clover and bring these species together as part of a common gene pool.

**Results:**

Ten verified F_1_ (6*x T. ambiguum* x 2*x T. occidentale*) hybrids were produced by embryo rescue and seven were studied further. All four investigated for chromosome number were 2*n* = 4*x* = 32 and FISH confirmed the expected 21 *T. ambiguum* and 8 *T. occidentale* chromosomes. Hybrid fertility was extremely low but 2*n* female gametes functioned with white clover pollen to produce seeds. Derived plants were confirmed using FISH and were successfully backcrossed to white clover to produce partially fertile breeding populations.

**Conclusions:**

Although *T. occidentale* and 6*x T. ambiguum* are widely separated by geography and ecological adaptation they have maintained enough genomic affinity to produce partially fertile hybrids. Inter-fertility of the hybrids with allotetraploid *T. repens* showed that *T. occidentale* can provide a genetic bridge between 6*x T. ambiguum* and white clover to produce plants with new phenotypes combining the traits of all three species. Use of this information should enable potentially valuable stress tolerance traits from 6*x T. ambiguum* to be used in white clover breeding for the first time.

## Background

The genus *Trifolium* has approximately 250 species distributed throughout Europe, Africa, W Asia and the Americas [1]. Within the genus, a small section (*Trifoliastrum*) of 14 species has been delineated by DNA sequence phylogeny [2]. The species in the section range from diploid annuals to hexaploid perennials, and are scattered separately across Europe and W Asia. Among other species, the section includes *T. ambiguum* (Caucasian clover), *T. occidentale* (western clover) and *T. repens* (white clover). Caucasian clover (*Trifolium ambiguum*) occurs as a polyploid series in continental W Asia centred around the Caucasus region. The polyploid series approximates an altitudinal gradient, with diploid populations at high altitudes, hexaploids at the lowest altitudes and tetraploids between [3]. This variation across the series suggests that the genomes of the ploidal forms carry adaptive genetic differences. In contrast, *T. occidentale* is a diploid, strictly maritime species confined to the gulf-stream sea coasts of W Europe from Portugal to Ireland, SW England and Wales [4–8]. *T. repens* is a tetraploid with a wide natural distribution, spanning Europe and W Asia, and is the most important of about 10–15 clover species used in world-wide agriculture [9].

Although white clover is widely used, it is a stoloniferous plant that moves across the soil surface and has a shallow, fibrous root system that makes it vulnerable to drought conditions. This vulnerability is exacerbated by the fact that white clover is also an inefficient user of water [10, 11]. By contrast, *T. ambiguum* is of considerable agricultural interest as a pasture legume because of its deep root system, underground creeping growth habit through rhizomes, drought tolerance, pest and disease resistance and high feed quality [9]. These attributes complement several shortcomings that restrict the performance of white clover on farms, especially in dry environments.

*T. repens* is of hybrid (allotetraploid) origin [12] and is unusual as the genus is remarkably lacking in reticulate speciation [2]. Despite the rarity of natural species crossing, several species pairs in Section *Trifoliastrum* have been artificially hybridized, mainly to add new genetic diversity to clover breeding populations [9]. These crosses included those between *T. repens* and artificial tetraploid *T. occidentale* [13–16]. Hybridization of 4*x T. ambiguum* and 4*x T. repens* has also been achieved, but with very low success rates and with considerable difficulty using embryo/ovule culture [17–19]. These hybrids have been difficult to breed and despite several generations of population development and field selection [9, 20, 21] have, to-date, produced only one cultivar. This might, in part, be due to the vegetative inferiority of the tetraploid form of *T. ambiguum* used, and this increases the desirability of finding ways to incorporate the hexaploid form.

So far, the hexaploid form has been of greatest agricultural value. This form is vegetatively much more robust than the diploid and tetraploid forms, with large leaves, tall habit and large underground stems and roots [3] and it has been successfully deployed for forage production in the mid-western USA [22]. A potential strategy to improve the drought performance of white clover could be to hybridize it with this vigorous form of Caucasian clover. However, direct hybridization would need to overcome potential barriers of geographic distance, diverse ecological adaptations and polyploid speciation. Although F_1_ hybrids between white clover and 6x *T. ambiguum* have been obtained by embryo rescue [23], no fertile F_1_ hybrids have so-far been reported, and so the desirable hexaploid form has not been incorporated into the wider gene-pool for clover breeding.

An alternative strategy could be to use the knowledge that white clover is a hybrid between ancestral forms of *T. pallescens* and *T. occidentale* [12] and that there has been successful hybridization of 2*x* and 4*x T. ambiguum* with 2*x T. occidentale*. This suggested that *T. occidentale* could be used as a genetic bridge between white clover and *T. ambiguum* [24, 25]. In the present case, this would require the generation of not only fertile hybrids between 6*x T. ambiguum* and *T. occidentale*, but also the ability to obtain hybrids of these with a third species, *T. repens*. If achieved, this hybridization would have implications, not only for speciation in the genus, but also for expanding the gene-pool for clover breeding.

We therefore undertook a study to see whether fertile hybrids between 6*x T. ambiguum* and 2*x T. occidentale* could be produced by embryo rescue. Here we provide experimental evidence that 6*x T. ambiguum* can form fertile hybrids with 2*x T. occidentale* and that the 4*x* F_1_ hybrid plants were inter-fertile with white clover. This hybridization spanned the potentially isolating barriers of continental-scale distances, diverse ecological adaptations, and polyploid speciation, and provided evidence of a recent pan-Europe-W Asia species radiation. Furthermore, the results reveal the commercial potential for *T. occidentale* to be used as a genetic bridge for including 6*x T. ambiguum* in the gene-pool to facilitate the breeding of improved cultivars of white clover.

## Results

Pollination of 6*x T. ambiguum* with 2*x T.occidentale* led to a change of the maternal standard petal colour from white to pink within 24–48 h. This was interpreted as evidence that fertilization had occurred. The frequency of florets developing pods was low (10–20%) and fewer than 1% produced detectable embryos. Dissection of hundreds of pods produced useful numbers of embryos that were green and at the early-torpedo stage. Eighteen of these were grown on agar plates into plantlets that were transferred to potting mix in the greenhouse. (A poor batch of medium led to losses of an unknown number during culture). Three months after transfer, 16 putative hybrid plants were growing in the greenhouse. Ten of these were verified as hybrids and are listed in Table 1 with their parentage. Six of these grew to maturity, flowered and three were studied in more detail (Table 1).

**Table 1:** Parentage of 10 verified hybrids between 6*x T. ambiguum* and 2*x T. occidentale*

Hybrid number	6*x T. ambiguum* female	2*x T. occidentale* male	Further investigation
5	Endura-13	48–17	Plant & progeny described
26	Endura-13	48–17	Died
29	Endura-16	44–16	Not studied
31	Endura-10	49–11	Flowered, seeds obtained
33	Endura-12	44–16	Plant & progeny described
34	Endura-18	48–17	Flowered, seeds obtained
36	Endura-13	48–17	Lost
51	Endura-10	49–11	Very large leaved and vigorous. Chromosome preparations confirmed 2n = 32. Not further studied.
140	Endura-13	59–16	Plant described, no seeds obtained
229	Endura-16	44–19	Flowered, progeny grown

Early observations were that the hybrids plants were large plants, vegetatively closer in size to *T. ambiguum* than to the diminutive *T. occidentale*. They were similar to *T. ambiguum* in leaf surface texture and flowering pattern. However, they had morphological characteristics from both the parent species (Table 2), being semi-prostrate or semi-erect, with robust horizontal stems with adventitious roots only at the basal nodes. Leaflets were large and pointed and flower heads resembled those of *T. ambiguum.* Detailed measurements on pot-grown mature plants of three hybrids confirmed that the morphology of the hybrids was closer in shape and size to *T. ambiguum* than to *T. occidentale* (Tables 3, 4). Petioles of the hybrids were approximately double the length of those of *T. occidentale* and the leaflets were elongated and similar in shape to the leaflets of *T. ambiguum*. Stem thickness, leaf thickness and numbers of leaves and growing points per plant were also closer to those of *T. ambiguum*, while plant spread was inferior to both parents. Dry weights of all plant parts, except inflorescences (heads), were inferior to both parent controls (Table 4). Inflorescence numbers and DWs were transgressively much higher than both parent species. Flowering was terminal, as in *T. ambiguum*, rather than axillary, as in *T. occidentale*, and so the flowering stems were determinate, dying back to the basal nodes after seed maturity.

**Table 2:** Vegetative and floral characteristics of 2*x T. occidentale*, 6*x T. ambiguum*, and three 6*x T. ambiguum* x 2*x* T. *occidentale* hybrid plants 5, 33 and 140

Plant	Spread	Growth habit	Nodal rooting	Leaf surface	Leaf colour	Flowering pattern
2*x T. occidentale*	St	Prostrate	All nodes	Glossy	Dark green	Indeterminate
6*x T. ambiguum*	Rz	Erect	Nil	Glaucous	Green	Determinate
Hybrid 5	Rz + SSt	Semi-prostrate	1–3 basal nodes	Glaucous	Green	Determinate
Hybrid 33	Rz + SSt	Semi-erect	1–3 basal nodes	Glaucous	Dark green	Determinate
Hybrid 140	Rz + SSt	Semi-erect	1–3 basal nodes	Glaucous	Light green	Determinate

*Rz* rhizomatous, *St* stoloniferous, *SSt* semi-stoloniferous (distal part above horizontal)

**Table 3:** Results from non-destructive measurements in spring of 2*x T. occidentale*, 6*x T. ambiguum* and three 6*x T. ambiguum* x 2*x T. occidentale* hybrids (5, 33 and 140). *denotes significant differences (*P* < 0.05)

	VGP	ST	PS	LPP	PL	LLS	LT	IN	DF	RPP	ORD	DLNR	RzPP	RzD
*T. occidentale* 2*x*	118	2.2	73	166	94	1.16	0.21	6	89	12	2.4	1.0	0.0	–
*T. ambiguum* 6*x*	14	3.3	144	43	255	1.87	0.18	0	–	12	4.0	3.1	6.5	1.8
Hybrid 5	35	3.2	47	56	204	2.16	0.16	29	86	18	4.1	2.4	4.0	1.5
Hybrid 33	27	3.2	46	59	181	1.81	0.17	26	87	15	3.8	2.7	0.8	1.3
Hybrid 140	24	3.3	33	46	194	1.89	0.18	23	83	17	4.0	2.3	1.5	0.7
LSD (0.05)	14.7*	0.41*	27.6*	26.0*	22.8*	0.30*	0.023*	8.8*	6.9	6.6	0.83*	0.26*	2.50*	0.60*
SEM	5.1	0.14	9.7	9.1	8.0	0.11	0.008	3.1	2.4	2.3	0.29	0.09	0.88	0.21

*VGP* Vegetative growing points per plant, *ST* Stem thickness (mm), *PS* Plant spread (cm), *LPP* leaves per plant, *PL* Petiole length (mm), *LLS* Leaflet shape (length/width), *LT* Leaf thickness (mm), *IN* Inflorescence number, *DF* Days to flowering, *RPP* Number of roots per plant, *ORD* Diameter of the oldest root (mm), *DLNR* Diameter of the largest new root (mm), *RzPP* Number of rhizomes per plant, *RZD* Rhizome diameter (mm)

**Table 4:** Mean values of morphological traits of *Trifolium ambiguum* (6*x*), *Trifolium occidentale* (2*x*) and three 6*x T. ambiguum* x 2*x T. occidentale* hybrids (5, 33 and 140) following destructive harvest in summer. *denotes significant differences (*P* < 0.05)

Genotype	LNPP	VGP	IN	DWH	DWRes	DWFP	DWRS	DWRZ	NNR
*T. occidentale* 2*x*	1112	276	2	29.6	5.3	0.02	4.3	0.0	All nodes
*T. ambiguum* 6*x*	198	34	17	36.3	7.2	2.68	43.1	12.9 (23%)	0
Hybrid 5	305	43	120	19.4	3.1	17.6	5.0	1.0 (17%)	1–3
Hybrid 33	339	34	120	15.1	2.8	13.3	4.5	0.9 (17%)	1–4
Hybrid 140	277	30	137	20.2	3.1	16.8	4.5	0.4 (8%)	1–4
LSD (0.05)	135.2*	48.7*	35.3*	7.45*	2.13*	4.95*	7.39*	3.23*	
SEM	47.4	17.0	12.4	2.61	0.75	1.74	2.59	1.13	

*LNPP* Leaf number per plant, *VGP* Number of vegetative growing points per plant, *IN* Inflorescence number, *DWH* Dry weight (DW) harvested (above cutting height, g), *DWRes* DW of residual stems after cutting (g), *DWAGP* DW of above-ground part (g), *DWFP* DW of floral parts (g), *DWRS* Dry weight of root system (g), *DWRZ* Dry weight of rhizomes (g, with % of total below ground DW), *NNR* Nodes with nodal roots (per stem)

Below ground measurements on mature plants grown in pots (Table 3) showed that the three hybrids studied had thick roots similar in diameter to *T. ambiguum*. Rhizomes were present in all three hybrids but were fewer in number and, in two cases, thinner than those of *T. ambiguum*. However, the dry weights of the roots and rhizomes were markedly lower than *T. ambiguum* (Table 4).

### Cytogenetic analyses of the F_1_ hybrids

**Fig. 1:**
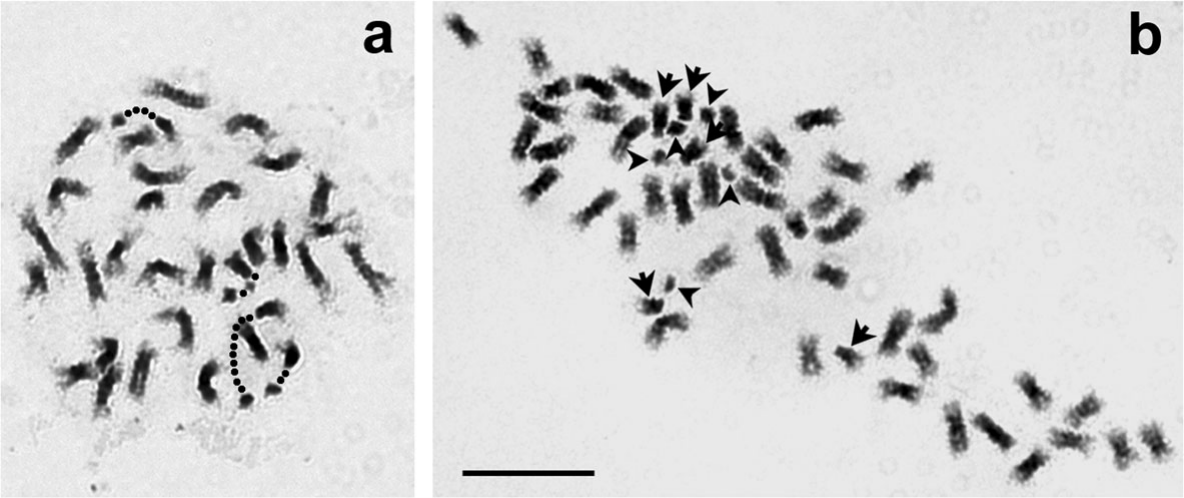
**a** Giemsa preparation in gray-scale of a somatic cell of Hybrid 5 (6*x T. ambiguum* x 2*x T. occidentale*) showing 2*n* = 4*x* = 32 chromosomes. Four NOR chromosomes are identified by dotted lines. **b** Giemsa preparation in grey scale of a somatic cell of an open-pollinated (OP) progeny plant of hybrid 5 (Hybrid 5OP-1). There are 2*n* = 48 chromosomes. Five NOR chromosomes are identified by arrows (main bodies) and arrowheads (satellites)

Somatic chromosome preparations of hybrids 5, 33, 34, 51 and 140 showed 2*n* = 4*x* = 32, each consistent with derivation from a *n* = 3*x* = 21 gamete from 6*x T. ambiguum* and a *n* = *x* = 7 gamete from *T. occidentale*. A cell of hybrid 5 is shown in Fig. 1a, revealing four satellited chromosomes and a vague indication of a 3:1 ratio in chromosome sizes.

**Fig. 2:**
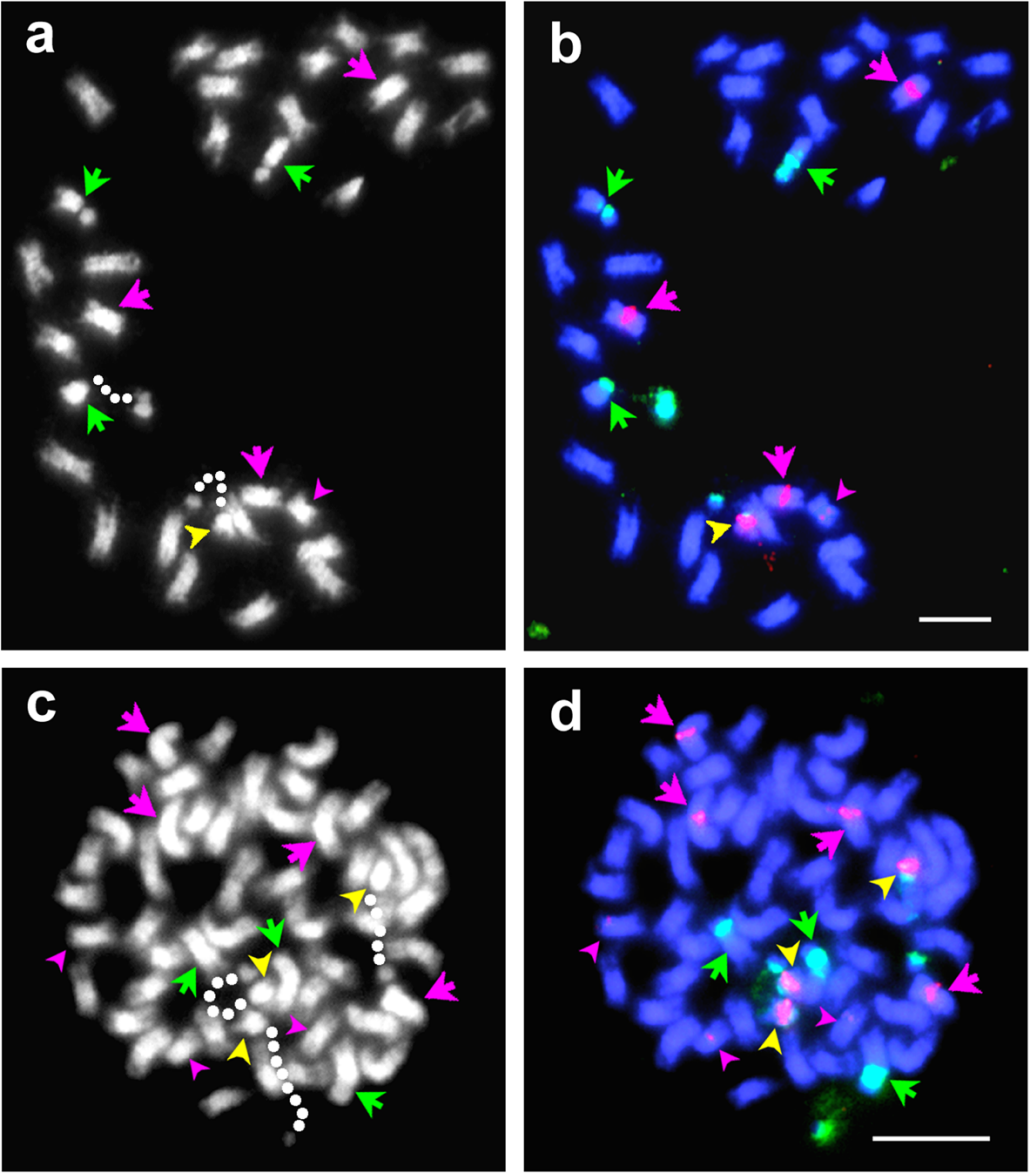
(**a**-**b**) A somatic cell of Hybrid 33 (6*x T. ambiguum* x 2*x T. occidentale*) showing 2*n* = 4*x* = 32 chromosomes. **a** DAPI stained cell in grey scale. Dotted guidelines represent decondensed NORs. **b** FISH on the same cell using 5S (pink) and 35S (green) rDNA probes. In both **a** and **b**, NOR chromosomes from *T. ambiguum* (green arrows) and *T. occidentale* (yellow arrowheads) are indicated. A red arrowhead identifies a *T. occidentale* chromosome with a minor 5S signal. In **b**, red arrows indicate *T. ambiguum* chromosomes with large 5S signals. **c**-**d** A somatic cell of an OP progeny plant of hybrid 33 (Hybrid 33OP-1). c DAPI stained cell in grey scale. Dotted guidelines represent decondensed NORs. **d** FISH on the same cell using 5S (pink) and 35S (green) rDNA probes. In both **c** and **d**, NOR chromosomes from *T. ambiguum* (green arrows) and *T. occidentale*/ *T. repens* (yellow arrowheads) are indicated. Red arrowheads identify *T. occidentale*/*T. repens* chromosomes with 5S signals. In **d**, red arrows indicate *T. ambiguum* chromosomes with large 5S signals

FISH using 35S and 5S rDNA as probes showed that the chromosome complement of hybrids 33 and 140 were consistent with expectations [26] following fusion of normal gametes from both species. Figures 2a, b show a somatic cell of hybrid 33. One NOR-bearing chromosome with a 5S signal on the opposite arm was from *T. occidentale* and three NOR-chromosomes lacking 5S signals were from *T. ambiguum*. There were three chromosomes with large 5S signals from *T. ambiguum* and one with a very small 5S signal from *T. occidentale*. The *T. occidentale*-derived NOR was observed to be always decondensed while one or two of the *T. ambiguum*-derived NORs were often, but not always, condensed.

### Meiotic configurations in F_1_ hybrids

Metaphase I meiotic configurations were analysed for hybrids 5 and 34 (Table 5). The results for both plants were similar, showing high numbers of univalents per cell, a few bivalents and trivalents and very few quadrivalents. Hybrid 5 was also observed at anaphase I where it showed frequent laggards and only infrequent 16–16 disjunction.

**Table 5:** Meiotic configurations at metaphase I in hybrids 5, 34 and 140

Plant		Meiotic configurations				Anaphase I disjunction
	No	I	II	III	IV	
	PMCs	$\overline{x}$ (range)	$\overline{x}$ (range)	$\overline{x}$ (range)	$\overline{x}$ (range)	
Hybrid 5	61	9.1 (6–13)	5.5 (3–8)	3.0 (1–4)	0.7 (0–1)	Only 2 cells 16–16, laggards
Hybrid 34	72	9.6 (3–14)	5.5 (3–9)	3.0 (1–6)	0.6 (0–2)	–

### Fertility of the F_1_ hybrids

Pollen fertility of the F_1_ hybrids was extremely low, with pollen staining ranging from 3 to 13% (Table 6). Based on the very low pollen fertility and unsuccessful attempts to obtain seed by hand pollination, cloned copies of six hybrids were grown in pots outside and were open-pollinated by bees. Surrounding plants included diverse genotypes of both parent species along with diploid and tetraploid *T. ambiguum* and white clover. Five of the hybrids produced seed from open-pollination. Seed-set was determined on four hybrids and ranged from zero to 14 seeds per 100 heads (i.e. up to approximately 0.28 seeds per 100 florets, assuming an average of 50 florets per head) (Table 6). Seed-lots from hybrids 5, 33 and 229 were germinated and the progeny were investigated.

**Table 6:** Male fertility (pollen stainability %) and female fertility (OP seed-set) of six hybrids between 6*x T. ambiguum* and 2*x T. occidentale*

		OP seed-set		
Hybrid	PS (%)	Nos seeds + small seeds	No Infls	Seeds/100 heads
5	9	4	ND	ND
31	3	15	ND	ND
33	2,4	6 + 4	150	4
		21 + 20	450	5
34	5	2 + 9	150	1
140	5	0	56	0
229	13	18	130	14

### Descriptions of OP progeny plants from hybrid 5

Hybrid 5 produced only one small normal OP seed and five very small seeds. Only the normal seed developed into a mature plant, designated 5OP-1. The seedling of 5OP-1 was raised in a greenhouse in winter and was abnormal, with a pale green unifoliolate leaf and a yellow first trifoliolate leaf. Subsequent trifoliolate leaves were pale green with very narrow elongate leaflets. The mature plant had very narrow leaflets with double-V white markings. The plant produced multiple short stems that were rooted at the basal nodes. Flower heads were *T. ambiguum*-like but were white rather than pink at the base. Mature foliage was light green while leaves produced in some seasons in an unheated greenhouse were yellow-green, suggesting sensitivity to cool temperatures.

5OP-1 was moderately fertile with pollen stainability of 35% and a somatic chromosome complement of 2*n* = 48 (Fig. 1b). The number of satellite chromosomes was five (Fig. 1b) as would be expected if white clover was the male parent.

### Descriptions of OP progeny plants from hybrid 33

Sixteen seeds harvested from hybrid 33 after OP were germinated and 12 mature plants were obtained. Several of these flowered and showed varying degrees of fertility as shown by pollen stainability and seed-set from controlled crosses (Table 7). There was strong phenotypic evidence that to form some of these progeny, hybrid 33 had been pollinated by white clover (Table 7). For example, progeny plants 33OP-1, 3, 13, 16, 18, 19, 20 all were stoloniferous and nodally rooted. Plants 33OP-13 and − 16 also carried purple leaf mark alleles from white clover. Progeny plant 33OP-17 was apparently rhizomatous, while several plants with horizontal stems and nodal roots only at the base could not be described as stoloniferous and might not have had a white clover parent. Plant 33OP-1 and its descendants were subjected to further study.

**Table 7:** Description of progeny from OP of hybrid 33

Plant number	Description^a^	PS^b^ %	Seed-set^c^	Fate
33OP-1	Stoloniferous Nodal roots 2*n* = 51	45	Self 43 seeds/19 infl	Used in breeding
			xRET 20 seeds/3 infl	
			OP 19 seeds/11 infl	
			RETx 30 seeds/3 infl	
33OP-3	Stoloniferous Nodal roots	11	ND	Died
33OP-11	Horizontal stems Basal nodal roots	31	Self 2 seeds/7 infl	Not used
33OP-12	Horizontal stems Damaged as seedling	2	ND	Not used
33OP-13	Stoloniferous Nodal roots R_l_		ND	Not used
33OP-14	Horizontal stems Basal nodal roots, short rhizomes 2*n* = ~ 56	37	Self 11 seeds/7 infl	Progeny obtained
			xRET 8 seeds/10 infl	
			xAMH 5 seeds/3 infl	
33OP-15	Horizontal stems Basal nodal roots	60	Self 1seed/3 infl xRET 1 sm seed/1 infl	Progeny obtained
33OP-16	Stoloniferous Nodal roots R_ld_	42	Self 3 seeds/2 infl xRET 13 seeds	Progeny obtained
33OP-17	Horizontal stems Rhizomes	–	–	Did not flower Died
33OP-18	Stoloniferous Nodal roots	2	ND	No progeny
33OP-19	Stoloniferous Nodal roots	7	Self 0 seeds/9 infl	No progeny
33OP-20	Stoloniferous Nodal roots Terminal flowering	8	Self 2 small seeds/10 infl	No progeny

^a^ R_l_, R_ld_ red leaf and diffuse red leaf phenotypes; ^b^PS, pollen staining; ^c^Seed sets: Self, after self-pollination; xRET, after pollination with *T. repens*; OP, after open pollination; RETx, after pollinating *T. repens*; xAMH, after pollination with 6*x T. ambiguum*; ND, not determined

Plant 33OP-1 was robust and stoloniferous with elongated leaves that were obtuse, rather than acute, at the tips. The leaves carried two white V markings and the texture was thinner and less stiff than that of 6*x T. ambiguum* and hybrid 33. Flowers produced in the first spring had a strong scent of coconut oil. The anthers were small, and possibly indehiscent and, while pollen was not abundant, it showed moderate stainability (Table 5). The plant was also partially female fertile, giving 1–4 seeds/inflorescence when both open-pollinated and self-pollinated.

Flow cytometry indicated that this plant was near-6*x*, suggestive of the union of a near-4*x* (i.e. unreduced) female gamete from hybrid 33 and a 2*x* = 16 male gamete. Somatic preparations confirmed that 33OP-1 had a chromosome complement of 2*n* = 51. FISH using 35S and 5S rDNA as probes (Fig. 2c, d) showed that, in addition to the full complement of marker chromosomes described above for hybrid 33, this plant had one more NOR-bearing chromosome with a 5S signal on the opposite arm, and a chromosome with a medium-sized 5S signal, as expected if white clover was the provider of the 2*x* = 16 male gamete [26]. In addition, there was a fourth, *T. ambiguum*-derived, chromosome carrying a large 5S signal.

### Seeds and progeny from hybrids 31, 34 and 229

OP seeds were obtained from all three of these hybrids and was placed in storage. Nineteen OP progeny plants from hybrid 229 were grown in pots in a greenhouse. Only a few plants thrived, generally showing low fertility. A few had nodally rooted stolons and rounded leaves consistent with having had white clover as the male parent.

### Progeny of 5OP-1 from controlled crosses

5OP-1 produced 3–4 seeds/head on self-pollination and 2–3 seeds/head when pollinated with two different white clover plants. One white clover head was pollinated with 5OP-1 and this resulted in 14 seeds.

Three seeds were germinated from the pollination of 5OP-1 with a white clover plant (BRI x WIL-1)-902, carrying the dominant *R*_*l*_ allele for purple leaf colour in heterozygous form. All three of the progeny showed white clover traits, being stoloniferous with nodal roots and axillary inflorescences and one had purple leaves, confirming the white clover paternity. A further five progeny plants were obtained after pollination of 5OP-1 with a second white clover plant and four of these were stoloniferous with nodal roots, again indicating successful fertilization by white clover. All eight plants obtained from crosses by white clover were partially fertile when open-pollinated among themselves, but seed-sets were low, the best two plants averaging approximately 10 and 14 seeds/head. Most of this seed was stored but a few (BC_1_F_2_) plants were grown and further backcrossed to white clover to produce putative BC_2_ seed that was also placed in storage.

### Progeny from controlled crosses of OP plants from hybrid 33

Plants 33OP-1, 11, 14, 15, 16 and 20 produced small numbers of seeds following self-pollination (Table 7). Plants 33OP-1, 14 and 16 were inter-fertile with white clover, and 33OP-14 also set seed following pollination by 6*x T. ambiguum*. The progeny of 33OP-1 were investigated further.

Self-pollination of 33OP-1 gave about 2 seeds/head (Table 7). Twenty self seeds were germinated and these gave nine mature plants, with the remainder having major abnormalities and not thriving. All of the survivors expressed predominantly *T. ambiguum* traits (elongated leaflets, semi-erect stems) but also had basal nodal roots, a *T. repens*/*T. occidentale* trait. Six were robust plants, with terminal inflorescences and low pollen staining (13–36%).

Hand-pollination of 33OP-1 by a white clover plant heterozygous for red and diffuse red leaf markings (*R*_*l*_/*R*_*ld*_) produced seeds that gave rise to verified progeny carrying one or the other leaf marking. Most of the progeny had pale green or variegated yellow/green leaflets, especially in the early seedling stages, and only about one third survived to maturity (25 seeds gave 22 seedlings of which eight grew to maturity and flowered). The mature plants were robust with thick, densely branched horizontal stems that lacked or had only basal nodal roots. Flow cytometry showed that they were near-5*x*. Chromosome counts on two of them confirmed numbers of 2*n* = 42 and 41–42. The latter of these plants, carrying a white V mark and the diffuse red leaf allele was used as the pollen parent on an unmarked white clover plant. Nine verified progeny were obtained from 10 seeds sown (one plant was a self on the white clover mother plant). All nine progeny were strong, stoloniferous, plants resembling white clover. Apart from one that failed to flower, they were moderately fertile with a mean pollen staining of 43% (range 29–69%) and none showed the pale green foliage sectors that had been characteristic of the previous generation. Flow cytometry estimates averaged 2*n* = 4.7 and ranged from 4.5–5.0. These plants were the products of at least two (probably three) crosses of hybrid 33 (AAAO) with white clover. Advanced generation progenies were produced for further investigation.

### Progeny from controlled crosses of OP plants from hybrid 229

One OP progeny plant from hybrid 229 (229 OP-2) was crossed as male with white clover and produced nine seeds from two inflorescences. Nine progeny plants were grown and eight produced some seeds after open pollination. These plants showed variable expression of nodal rooting of the horizontal stems, ranging from no roots to strong rooting at all nodes.

## Discussion

This research has shown that 6*x T. ambiguum* could be crossed with 2*x T. occidentale* to produce partially fertile 4*x* hybrids that were inter-fertile with 4*x* white clover (*T. repens*). These results have implications for both species evolution and potential introgression breeding of white clover. The fact that white clover is involved, implies potential for combining the agronomicaly desirable features of both species into new hybrid populations, perhaps leading to introgression of 6*x T. ambiguum* genetics into white clover.

Previous studies [24, 25] have shown that both 2*x* and 4*x T. ambiguum* could be hybridized with 2*x T. occidentale* and that the F_1_s were inter-fertile with white clover. This indicated that all four taxa were derived from a common ancestor and were part of a species radiation that now covers all of Europe and parts of W Asia. This radiation involved polyploidization and led to extremely divergent morphologies and ecological adaptations across a very wide geographic region. For both 2*x* and 4*x T. ambiguum* and *T. occidentale* it was apparent that the evolution of extremely divergent species had occurred in the absence of strong barriers to hybridization and potential gene flow. This study has shown that 6*x T. ambiguum* also shows these properties, despite further polyploidization and adaptational divergence.

Somatic chromosome counts on several F_1_s gave 2*n* = 4*x* = 32, confirming the expected number. FISH using 5S and 35S rDNA probes, carried out on two of the F_1_s also confirmed a ratio of three *T. ambiguum* to one *T. occidentale* sub-genomes, based on characterization of the parents [26].

Observations indicated no evidence of major pre-fertilization barriers when 6*x T. ambiguum* was pollinated by *T. occidentale*. Post-fertilization, the frequency of rescuable embryos was low but green torpedo embryos were not rare either, and so dissection of a few hundred ovules was rewarding. Most of the dissected embryos grew into large, robust plants that were vegetatively more like *T. ambiguum* in leaf shape and in having rhizomes, but they also weakly expressed basal nodal rooting, reflecting *T. occidentale* parentage. In general, the phenotypes of the F_1_ plants were consistent with the 3:1 ratio of *T. ambiguum* to *T. occidentale* sub-genomes. However, clear exceptions were plant spread, which was poorer than in either parent, and inflorescence development, which was positively transgressive.

Despite the impressive vegetative and floral vigour of the F_1_s, both male fertility (pollen staining) and female fertility (seed-set) were extremely low. Of six hybrids checked, five were below 10% in pollen staining. Very low pollen staining (< 3%) was also observed in three F_1_ hybrids between 2*x T. ambiguum* and 2*x T.occidentale* [24] and, similarly, it was approximately 9% in one 3*x* F_1_ between 4*x T. ambiguum* and 2*x T. occidentale* [25]. Female fertility (OP seed-set) was, however, apparently much lower in the 6*x T. ambiguum* x 2*x T. occidentale* F_1_s than in the hybrids involving 2*x* and 4*x T. ambiguum*. One 2*x* hybrid produced nearly one (0.8) OP seeds/head [24], while the 3*x* hybrid produced 1–2 OP seeds/head [25]. In contrast, the seed-sets obtained in the present study ranged from 0.00–0.14 seeds/head (Table 4). The very low fertilities were consistent with an expected low frequency of genomically balanced gametes following meiosis in these plants with uneven numbers of parental sub-genomes.

Meiotic analyses of two of the F_1_s provide a probable explanation for the low fertility. Data for hybrids 5 and 34 (which both produced progeny) indicated very dysfunctional meiosis, with more than half of the chromosomes per cell, on average, either unpaired (univalent) or involved in trivalents (Table 5). Unequal disjunction of these, as was observed, was likely to lead to a predominance of reduced gametes with unbalanced chromosome compositions. However, an average of one quadrivalent every two cells suggested that some A-O pairing had occurred, potentially leading to genomic recombination.

Three of the OP progeny of hybrids 5 and 33 (5OP-1, 33OP-1, 14) were checked for chromosome number and, in all cases, they were derived through the functioning of unreduced hybrid female gametes. In two cases (5OP-1 2*n* = 48, 33OP-1 2*n* = 51) the male gamete was from white clover which reliably produces gametes with 16 chromosomes. Therefore, the female gametes had, respectively, 32 and 35 chromosomes, both close to the expected unreduced number of 32. Although the gamete from 5OP-1 could have been a fully balanced 2*n* gamete, it is possible, given the meiotic abnormalities, that it was also unbalanced and coincidentally 2*n* = 32. The aneuploid 2*n* gamete from hybrid 33 was found to have an additional 5S rDNA chromosome from *T. ambiguum*. This gamete could have had a 4*x* + 3 constitution, but other chromosome imbalances were also possible. The third case (33OP-14, 2*n* = ~ 56) was also consistent with derivation from a near-2*n* hybrid female gamete. These findings suggest that 2*n* gametes were the predominant (and perhaps only) class of functional gametes produced by these AAAO hybrids. The frequencies of seeds set were very low (e.g. hybrid 33, 0.8–1.0 seeds per 1000 florets). Assuming 1–3 ovules per floret, and that only 2*n* gametes were fertilized, this would produce a frequency range of successful 2*n* female gametes of 0.03–0.10%. Because some 2*n* eggs would have been unfertilized, this probably underestimated the frequency of 2*n* gametes, but it nevertheless falls within the known range of 2*n* female gamete production and at the low end for hybrids [27]. The results are therefore consistent with unreduced gametes being the predominant or only class of fertile gametes from the AAAO hybrids. Unreduced gametes were also observed to have functioned in the progeny of 2*x T. ambiguum* x 2*x T. occidentale* (AO) F_1_ hybrids [24].

Among six F_1_ hybrids that were investigated for fertility, five proved to be partially fertile and produced OP progeny (Table 4). The three progeny families that were grown were also fertile and produced progeny from further crosses. In one case (33OP-1) the male parent was confirmed by FISH to have been white clover. This plant proved to be inter-fertile with white clover – thus establishing a tri-species lineage involving 6*x T. ambiguum*, 2*x T. occidentale* and 4*x T. repens*. While initially aneuploid, this lineage retained fertility when taken through subsequent generations. The progeny of hybrids 5 and 229 also were inter-fertile with white clover and thus also established tri-species lineages.

The implications of these lineages are two-fold. First, they establish that these three taxa, one 2*x*, one 4*x* and one 6*x* form an inter-related group derived from a recent, probably diploid, common ancestor. Second, from a clover breeding viewpoint, they show that *T. occidentale* can be used as a genetic bridge to attempt to supplement the white clover gene-pool by introducing genomic constituents from 6*x T. ambiguum*, which is generally regarded as the most agronomically suitable form of this species.

Speciation has involved polyploidization along with marked phenotypic and adaptive divergence. *T. occidentale* is stoloniferous and adapted to saline, coastal habitats and 6*x T. ambiguum* is rhizomatous, and adapted to high altitude continental habitats. *T. repens* is an allotetraploid derivative of hybridization between alpine *T. pallescens* and coastal *T. occidentale* and its adaptation spans the habitat range of the whole group. Previous work [24, 25] showed that 2*x* and 4*x* forms of *T. ambiguum* formed fertile hybrids with *T. occidentale*, also after embryo rescue. These inter-fertilities suggested that species divergence had probably not occurred from an accumulation of genetic changes in inter-fertility but rather that such changes had occurred later after the separation and independent development of the ancestral populations [24, 25].

However, genetic barriers to hybridization of 6*x T. ambiguum* with both *T. occidentale* and *T. repens*, although incomplete, appear to be quite strongly developed. With *T. occidentale*, embryos were few and developed only to the early green torpedo stage and had to be rescued. Although most of the F_1_ hybrids were robust, their fertilities were extremely low. Only unreduced gametes appeared to be functional with white clover and the tri-species progeny plants were near-6*x* and were generally also low in fertility. Based on this small sample, it appears that the polyploidization processes that led to the formation of 6*x T. ambiguum* have been accompanied by accumulated genetic changes that have reduced, but not eliminated, inter-fertility with the sister taxa.

From a clover breeding viewpoint, this group, which includes other species, has been referred to as the “white clover complex” [28] and is a group of *Trifolium* species phylogenetically closest to white clover (*T. repens*). This complex is defined by presence in section *Trifoliastrum* [2] and ability to cross-hybridize with at least one other species in the group. Williams [18] further subdivided the species according to the ease of crossing with white clover. 4*x T. ambiguum* fell into the tertiary gene pool (requiring embryo rescue) while 2*x* and 6*x T. ambiguum* were placed in the quaternary gene pool (requiring both bridge crossing and embryo rescue). Infertile hybrids between 6*x T. ambiguum* and *T. repens* have been reported [23]. If these hybrids subsequently prove to be partially fertile, then 6*x T. ambiguum* could be moved to the tertiary gene pool.

**Fig. 3:**
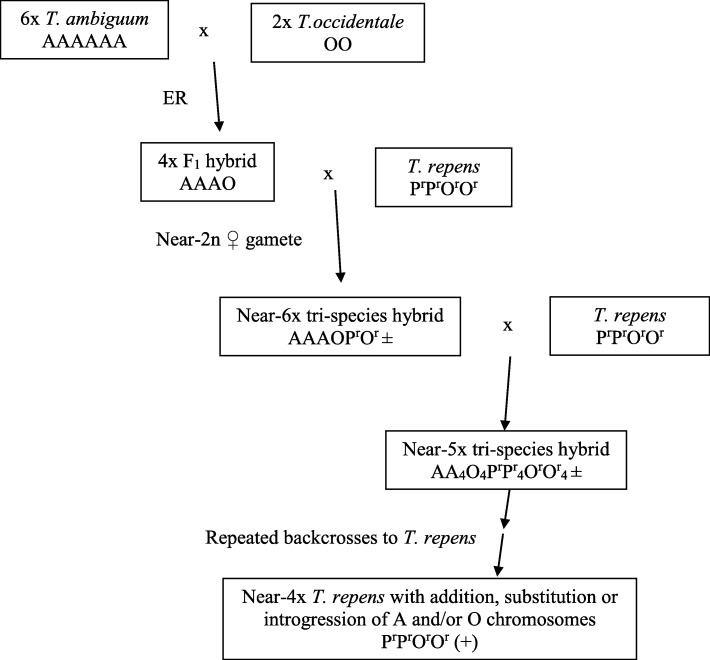
Crossing plan used to incorporate 6*x T. ambiguum* into the *T. repens* breeding pool. Abbreviations: ER embryo rescue, A, A_4_, O, O_4_ expected full (*x* = 8) or half *T. ambiguum* or *T. occidentale* sub-genomes, P^r^, P^r^_4_, O^r^, O^r^_4_ expected full or half ancestral *T. pallescens* and *T. occidentale* sub-genomes from *T. repens*

The breeding plan to incorporate 6*x T. ambiguum* into the *T. repens* breeding pool using *T. occidentale* as a bridge species is given in Fig. 3. As an example, where hybrid 33 was the F_1_ (AAAO), one near-6*x* progeny plant from pollination with *T. repens* [P^r^P^r^O^r^O^r^] was 33OP-1 (2*n* = 51). This was derived from a near-4*x* (*n* = 35) female gamete and a normal (*n* = 2*x* = 16) white clover gamete. Further backcrosses to *T. repens* led to progressive reductions in ploidy towards near-4*x T. repens* and these would have carried chromosome additions, deletions and substitutions from 6*x T. ambiguum* and *T. occidentale*. The presence of quadrivalents at meiosis in the F_1_ hybrids indicated the possibility of recombination between 6*x T. ambiguum* and *T. occidentale* chromosomes, as previously reported for hybrids involving 2*x* and 4*x T. ambiguum* and *T. occidentale* [24, 25]. In the present case, should there have been any interspecific chromosome pairing followed by recombination at any meiosis in the breeding process then *T. occidentale* could function as a genetic bridge, potentially leading to introgressions from 6*x T. ambiguum* into *T. repens* backcross families.

This breeding plan indicated that significant levels of aneuploidy and mixed, unbalanced sub-genomes would lead to unpredictable genomic constitutions in the intermediate generations. In practice, the early backcross derivatives of 33 OP-1, although aneuploid, were robust plants with moderate fertilities. More advanced generations were produced in good numbers and will be investigated for evidence of addition, substitution or introgression from 6*x T. ambiguum* into white clover. Success would mean that the desirable features of 6*x T. ambiguum* that are absent from the white clover primary genepool would become available for future breeding and selection and potentially improve the performance of white clover populations in marginal, especially dry, environments.

## Conclusions

Diploid *T. occidentale* and hexaploid *T. ambiguum* have maintained enough genomic affinity to produce partially fertile hybrids despite widely divergent geographic distributions and ecological adaptations. Inter-fertility of the hybrids with allotetraploid *T. repens* showed that *T. occidentale* can provide a genetic bridge between 6*x T. ambiguum* and white clover to produce plants with new phenotypes combining the traits of all three taxa. Use of this information should enable potentially valuable stress tolerance traits from 6*x T. ambiguum* to be used in white clover breeding.

## Methods

### Plant materials

All living plants used in this study were derived from cultivars and accessions conserved in long-term storage in the Margot Forde Forage Germplasm Centre, AgResearch Grasslands Research Centre, Palmerston North, New Zealand. Five hexaploid (6*x*) *T. ambiguum* plants derived from self-pollination of a single plant of the commercially available cv. Endura (PGG-Wrightson Seeds, Christchurch, New Zealand) were used as female parents (designated Endura self-10, 12, 13, 16, 18). *T. occidentale* seeds (accession code OCD) were made available for research purposes only and were originally from collections made at sea level sites in N Spain under an approved Agreement for the Acquisition of Material for Plant Genetic Resources. Five derived plants were used as male parents: plant 44–16 (accession OCD 1157 Praia de Lorenzo), plant 48–17 (OCD 1162 Faro de Cabo Villano), plants 49–4, 49–11 (OCD 1163 Camarinas), and plant 59–16 (OCD 1172 Playa de San Antolin). Verifications of species identifications were made by the senior author.

### Hybridization and embryo rescue

The plants were brought to flower in an insect-free greenhouse at temperatures of 15–28 °C and a photoperiod of 16 h. Crossing was done by hand by placing pollen from *T. occidentale* on the exposed stigmas of *T. ambiguum* using the method of Williams [29]. After 8–10 days, early-torpedo embryos were taken from the ovules and placed under aseptic conditions on CR7 medium [30] in petri dishes. Approximately 4 weeks later, the plantlets were transferred to CR5 (root initiation medium) [30]. After a further 8–12 weeks the plants were potted into a peat/sand medium in the greenhouse. Hybridity was confirmed using the isozymes SDH and PGI [31], DNA sequencing of the nuclear ITS and chloroplast TrnL intron regions [2] and by fluorescence in situ hybridization (FISH) [26].

### Plant propagation, fertility assessment and progeny development

Mature plants were clonally propagated by cuttings. Flowering plants were placed outside for a full seasonal cycle to be open-pollinated (OP) by bees in the presence of the parent species and white clover.

Male fertility was estimated from pollen staining (Table 1) and determined by extracting mature pollen, staining with 1% acetocarmine and counting the percentage of full stained grains among a minimum of 300 at 200x magnification. Female fertility was assessed using seed-set (numbers of seeds per head) following self- or cross-pollination.

### Phenotypic descriptions

Three interspecific hybrids were characterized, along with plants of 6*x T. ambiguum* (“Endura”) and 2*x T. occidentale* (OCD 1168–14) in a pot experiment in a greenhouse at the Grasslands Research Centre, Palmerston North. The design, conduct and analysis of the experiment were as previously described [25]. Cuttings were planted in July 2008 and measurements were taken non-destructively in the next spring (October 2008) when the plants were flowering, and then destructively in the following summer (January 2009). The qualitative comparisons made are listed in Table 2 and the quantitative traits recorded are listed in Table 3 (spring) and Table 4 (summer).

### DNA analysis, cytology and molecular cytogenetics

Total DNA preparation, PCR amplification and DNA sequencing of ITS and cpDNA regions were carried out as previously described [2]. The method for ploidal estimation by flow cytometry was described in [24]. Conventional meiotic analyses were carried out on PMCs from floral buds treated with alcoholic HCl carmine [32]. Somatic chromosome preparations used the flame drying technique [26] on actively growing root tips macerated with proteolytic enzymes. These preparations were stained by Giemsa for conventional mitotic analysis and were also used for FISH. Meiotic FISH preparations were made following enzymatic maceration of floral buds. The DNA probes and procedures used to identify the chromosomal locations of 5S rDNA and 35S rDNA sequences were described in [25]. The hybridization and post-hybridization procedures were as described by [26].

## Data Availability

The data sets from this study are available from the corresponding author.

## Abbreviations

2*n* gamete: Unreduced gamete
2*x*: Diploid
4*x*: Tetraploid
6*x*: Hexaploid
A: A full subgenome (8 chromosomes) of *T. ambiguum*
A_4_: A part subgenome (4 chromosomes) of *T. ambiguum*
BC: Backcross
DAPI: 4′,6-diamidino-2-phenylindole
F_1_, F_2_: First and second hybrid generations
FISH: Fluorescence in situ hybridization
ITS: Internal transcribed spacer
NOR: Nucleolus organising region
O: A full subgenome (8 chromosomes) of *T. occidentale*
O_4_: A part subgenome (4 chromosomes) of *T. occidentale*
OP: Open pollinated
O^r^: An ancestral *T. occidentale* subgenome derived from *T. repens*
PCR: Polymerase chain reaction
PMC: Pollen mother cell
P^r^: An ancestral *T. pallescens* subgenome derived from *T. repens*
rDNA: Ribosomal DNA
